# In Vitro Anatomical Models for Nasal Drug Delivery

**DOI:** 10.3390/pharmaceutics14071353

**Published:** 2022-06-26

**Authors:** Gerallt Williams, Julie D. Suman

**Affiliations:** 1 Aptar Pharma, 27100 Le Vaudreuil, France; gerallt.williams@aptar.com; 2 Next Breath, an Aptar Pharma Company, Baltimore, MD 21227, USA

**Keywords:** nasal cast, deposition, nasal spray, anatomical model, targeted delivery, drug delivery

## Abstract

Nasal drug delivery has been utilized for locally acting diseases for decades. The nose is also a portal to the systemic circulation and central nervous system (CNS). In the age of SARS-CoV2, the development of nasal sprays for vaccination and prophylaxis of respiratory diseases is increasing. As the number of nasal drug delivery applications continue to grow, the role of targeted regional deposition in the nose has become a factor is nasal drug development. In vitro tools such as nasal casts help facilitate formulation and product development. Nasal deposition has been shown to be linked to pharmacokinetic outcomes. Developing an understanding of the complex nasal anatomy and intersubject variability can lead to a better understanding of where the drug will deposit. Nasal casts, which are replicas of the human nasal cavity, have evolved from models made from cadavers to complex 3D printed replicas. They can be segmented into regions of interest for quantification of deposition and different techniques have been utilized to quantify deposition. Incorporating a nasal cast program into development can help differentiate formulations or physical forms such as nasal powder versus a liquid. Nasal casts can also help develop instructions for patient use to ensure deposition in the target deposition site. However, regardless of the technique used, this in vitro tool should be validated to ensure the results reflect the in vivo situation. In silico, CFD simulation or other new developments may in future, with suitable validation, present additional approaches to current modelling, although the complexity and wide degree of variability in nasal anatomy will remain a challenge. Nonetheless, nasal anatomical models will serve as effective tools for improving the understanding of nasal drug delivery.

## 1. Introduction

Nasal drug delivery has existed for many decades and is a common route of administration for local acting therapies for diseases such as allergic rhinitis, sinusitis, and congestion, and it is increasingly being used and explored for systemic delivery, e.g., migraine, pain management etc. [1]. Looking forward, there is strong interest in developing new treatments for CNS diseases such as Alzheimer’s and Parkinson’s by nasal administration.

A large number of factors influence the delivery of therapeutic agents via nasal sprays or aerosols, and during the investigating of new nasal therapies, in vitro testing is often used to guide research and development of such nasal products.

In vitro anatomical models of nasal cavities are widely employed to study the intranasal deposition of nasally administered therapies [2]. This approach serves as an in vitro tool that mimics human nasal cavity anatomy. This review article will highlight the evolution of nasal casts and how they are employed to support pharmaceutical development.

## 2. Complex Nasal Anatomy

**Figure 1:**
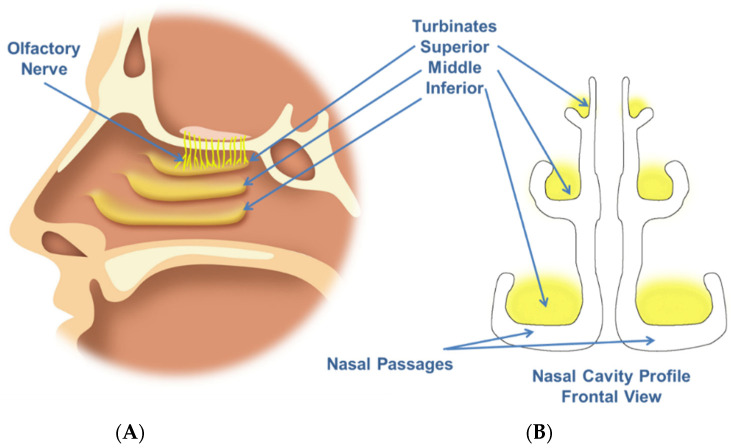
(**A**) Nasal cavity contains three turbinates that increase the surface area for warming and humidification of incoming air. Olfactory neurons innervate the superior nasal cavity. The turbinates and olfactory regions are nasal drug delivery targets. (**B**) The nasal passages are narrow and convoluted due to the turbinate structure. The complex structure and changing airflow through the nasal cavity may prevent deposition in the target area.

The human nasal cavity is known to be a complex structure (Figure 1), with specific anatomical regions that may be targeted depending on the intended therapeutic application. For example, the olfactory zone may be a target for CNS drug delivery.

Current regulatory guidance recommends that several in vitro tests (emitted dose, droplet or particle size distribution, spray pattern, plume geometry) can be used to characterize or compare nasal drug products in support of regulatory applications for new drugs or for generic drugs [3,4]. Although these in vitro methods are useful for informing issues related to reliability or manufacturing quality of nasal products, they provide limited useful information with regard to nasal deposition. Using nasal casts for this kind of in vitro approach allows for inexpensive and rapid investigations into formulation and device design prior to more expensive and complicated in vivo studies in humans. It should be noted the nasal cast studies are not a regulatory requirement. However, the in vitro deposition results may assist with justifying further development.

Precisely targeting the nasal spray deposition to specific regions of interest in the nasal cavity could improve local, systemic, and CNS drug delivery. Historically, this has proven to be difficult due to the complex anatomy of the nasal cavity. It is also known that nasal cavity anatomy and dimensions vary between individuals based on differences in their age, sex, ethnicity and disease state [5]. Furthermore, the anatomy is dynamic, with changing dimensions during normal inspiration/expiration and with the nasal cycle [2] and mucociliary clearance.

**Figure 2:**
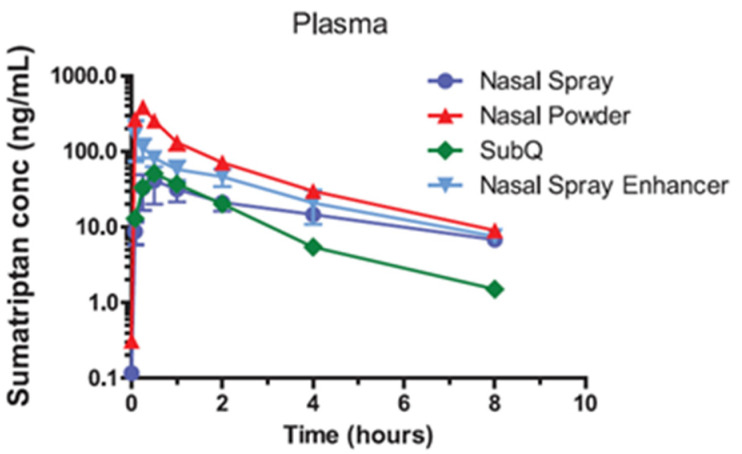
Pharmacokinetic profiles in NHP of sumatriptan when delivered as a nasal liquid spray, a nasal powder, a sub-cutaneous injection, and aqueous nasal spray with permeation enhancer [7].

The influence of intranasal drug deposition has been shown to have a significant impact on the efficiency of drug treatments, often resulting in different pharmacokinetic profiles for different drugs [6]. Studies where sumatriptan was delivered to the nasal cavity of non-human primates (NHP) in powder or liquid form, with different aerosol properties and therefore deposition, have shown that these different forms can influence the pharmacokinetic profiles (see Figure 2) [7].

**Figure 3:**
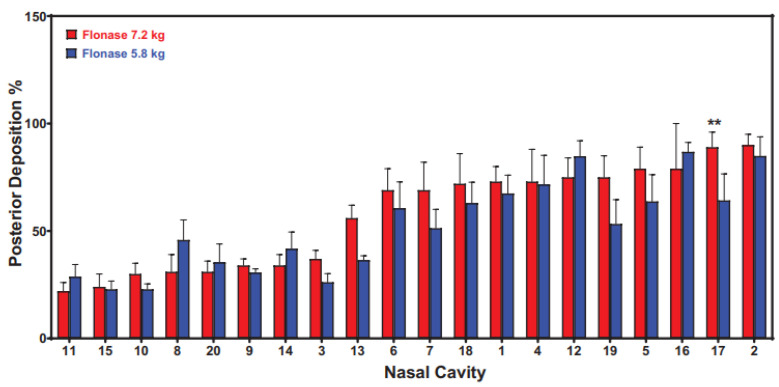
Posterior deposition in 20 nasal models sorted in ascending order. Two actuation forces were utilized. ** indicates significant difference in one model between the 5.8 and 7.2 kg actuation forces [8].

Deposition of locally acting nasal sprays may also differ depending on the patient’s anatomy. An in vitro deposition study using 20 individual models demonstrated differences in posterior deposition for a locally acting corticosteroid [8]. As can be seen Figure 3, the percent deposition ranges from ~25% to ~100%. The impact of individual differences has implications for design of clinical trials, bioequivalence, as well as relevant in vitro metrics used to characterize the drug product.

Intranasal drug delivery development requires the precise assessment of differences in drug deposition by comparing different delivery approaches including formulations and devices. Although in vivo evaluation in humans is clearly preferred, inexpensive and rapid in vitro evaluation with nasal cast models makes cast models an attractive approach for the evaluation of new nasal delivery products [9]; nevertheless, in vivo evaluation in humans is ultimately needed.

## 3. Evolution of Nasal Casts

Up to thirty-eight different nasal anatomical models have recently been reported in the literature [2,10,11,12,13,14,15]. They range in complexity from ‘simple’ geometries to ‘sophisticated incomplete’ geometries, ‘sophisticated complete’ geometries, and finally ‘human like’ geometry.

The first laboratory investigations of in vitro nasal characteristics employed cadaver heads, which on first thoughts could be considered as in vitro models which would closely represent human nasal geometry [16]. However, preservation issues and tissue retractions can significantly limit the uses of cadaver models. In order to overcome these limitations, plastination is regularly employed whereby water and lipids are replaced by silicone; the resulting anatomical structures are much more representative and can also be preserved. Such models have been validated in terms of nasal resistance by rhinometry [17] and have proven to result in anatomically complex and representative in vitro tools.

Today, 3D-printed models have made nasal casts more mainstream. Since the first 3D-printed models were developed [18], this in vitro approach has become more realistic and representative. Construction of the nasal casts often involves collecting raw data from individuals in 3D images, which are then digitally converted to be compatible with 3D-printing software. CT and MRI images are often used as the starting source. Various 3D-printing techniques are employed to build the physical nasal models from various polymer or plastic materials [19]. In addition, 3D-printing processes continue to evolve, allowing more precise models to be built [20].

Various publications have examined whether current models of nasal casts are realistic or relevant with regard to characterizing intranasal drug delivery. One such study [21] compared the only commercially available nasal cast, (Koken, Japan, primarily an educational tool) to a more accurately produced nasal geometry cast which was not commercially available. The study concluded that the Koken cast dimensions are larger than the normal anatomical range and that the drug deposition studies using this cast have doubtful validity. This finding was partly due to the Koken cast being produced from a cadaver, and the nasal passages of the cast were more patent compared with a cast made from a living model. It is, therefore, important to consider the source material and production process when selecting a model.

**Figure 4:**
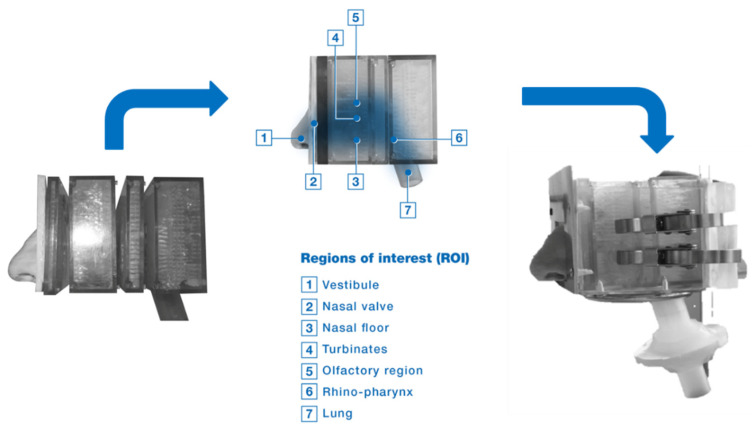
Example in vitro cast (APTAR/DTF/Univ. of Tours) and representative sections. The nasal cast is derived from adult male computed tomography (CT) scans. The nasal cast is constructed of epoxy plastic and divided into four segments.

In order to study the distribution of the deposited aerosol in different regions of the nasal models [22], disassemblable segmented models have been developed. Depending on the specificity needed, the nasal cast model can be divided into several anatomically relevant regions, or regions of interest (ROI). The areas of interest frequently studied are the following: nostrils, vestibule, turbinates, olfactory region and nasopharynx (Figure 4). The regions beyond the nasopharynx located at the back of the nasal casts can allow for the estimation of small inhalable particles, <10 µm, that may pass inadvertently to the lungs.

Further improvements to these sophisticated anatomical models in order to more closely mimic human nasal cavities have resulted in the use of artificial mucus or coatings, humidification and flexible nose segments [20]. The addition of coatings mimics the presence of a mucus layer and can more accurately represent the entrapment of droplets or particles in a humid nasal cavity [18,23]. In addition, coatings are required to eliminate particle bounce when evaluating nasal powders.

Numerous methods are employed to measure nasal deposition within the anatomic models, some of which are also used during in vivo evaluations. Various approaches including tracers such as colorimetric methods [13], fluorescein, and HPLC analysis for specific substances or drugs have been used to quantify deposition from nasal casts. Gamma scintigraphy, which is often used on nasal casts, has been used for qualitative or quantitative measurements. Radiotracer quantification can assess total or regional nasal deposition [24] but does have limitations for precise quantification in small ROIs.

Many nasal cast models are derived from individual or single cavities and these have some limitations. Alternative approaches [25] that propose an idealized nasal geometry which replicates average deposition for a population of subjects have been reported. Using large computational fluid dynamics (CFD) datasets, one can develop an idealized nasal geometry that replicates average regional deposition over the range of the dataset. Experiments with these kinds of idealized geometry replicated average deposition in the different anatomical nasal regions in comparison with both realistic replicas and previously published in vivo gamma scintigraphy data obtained utilizing commercial nasal spray products.

There is also interest in having specific models for particular populations such as pediatric, ethnic or geriatric populations. An example of such a cast is the Sophia model [26] in which an anatomically correct model of the upper airways of a young child was constructed. The model was selected to include the airway from the nasal cavity down to the subglottic region and allows for in vitro studies of aerosol deposition in young children.

In addition, models have been constructed which represent premature infant nose throat-models such as the PrINT-Model [27]; this is an upper airway model corresponding to a premature infant of 32-week gestational age and has been successfully used to predict airway deposition in this class of patients.

In vitro anatomical models can be used in conjunction with cell culture models in order to gain insights into more biological aspects such as permeation, mucoadhesion, ciliary clearance and/or cell toxicity, etc. In vitro cell culture models can come from both primary and immortalized cell sources [19]. Primary cells are cells collected from a donor and then cultivated using in vitro cell culture conditions and have the advantage of closely representing selected nasal tissue types (e.g., turbinate or olfactory mucosa) for specific studies related to these regions of interest. Some drawbacks of using such cells are low cell differentiation, the limited number of subcultures possible and a greater risk of contamination (e.g., bacterial or fungal).

An alternative to primary cell culture is the use of immortalized cells. They have advantages such as better standardization, ease of use in routine in vitro testing and are easily amplified and cultured. Standardized cell culture model examples include RPMI 2650, Calu-3 and 16HBE [19]. Both cell culture models offer advantages with the standardized immortalized cells often used in early work investigating drugs, excipients and formulations. These cells can then be employed when the nasal formulations are more advanced and more information can be ascertained about their performance in contact with true nasal mucosa.

## 4. Cast Applications

Nasal casts play a role in differentiating nasal delivery systems. Figure 4 highlights regional differences in deposition between a multi-dose nasal spray and nasal powder [22]. The nasal powder achieved approximately 30–40% deposition in the middle turbinate and olfactory regions. This highlights a potential advantage of nasally administered powders in targeted CNS delivery.

**Figure 5:**
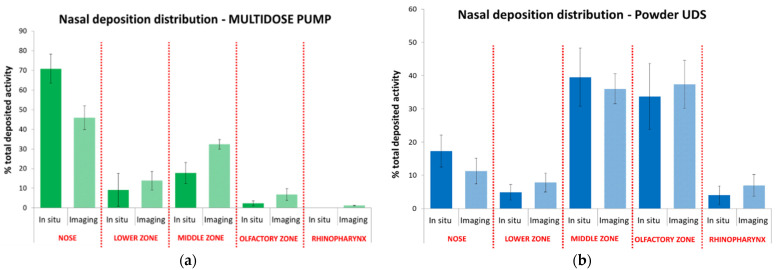
Percent deposition from a multi-dose aqueous nasal spray (**a**) and a unit dose (UDS) nasal powder (**b**). Deposition in the nasal cast trended with in vivo deposition as measured by gamma scintigraphy. (Dark green/Dark blue = in situ deposition with Fluorescein, Light green/Light blue = deposition by gamma scintigraphy).

In situ deposition from fluorescein spiked formulations was also compared to in vivo deposition using gamma scintigraphy (Figure 5). The results indicate that the nasal cast is a suitable surrogate for quantifying regional deposition.

**Figure 6:**
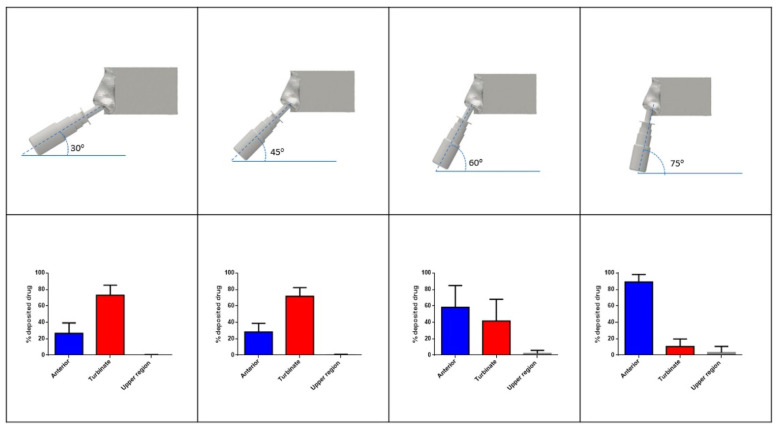
Percent nasal deposition after administration of 0.8% hypermellose cromolyn sodium nasal spray (*n* = 10). Deposition was evaluated after dosing using four different administration angles. The nasal cast was segmented to highlight deposition in three regions of the nose: anterior (blue), turbinate region (red), and upper region (grey) [28].

Historically, the instructions for use for nasal drug products have been vague. In the age of targeted nasal drug delivery, it is becoming clear that more specific instructions for use are required. In addition to human factors studies, nasal cast studies can play a role in defining patient instructions. The deposition angle from an aqueous nasal spray was evaluated in a nasal cast model by Warnken, et al. [28]. The study demonstrated that an administration angle between 30 and 45° increased deposition in the middle regions of the nose (Figure 6).

The relationship between inspiratory airflow and aerosol deposition patterns in in vitro nasal models is somewhat unclear. While some studies have concluded that there is no or very little effect of the airflow on nasal deposition [29], others studies have found deeper deposition beyond the nasal valve when increasing the airflow rates [30]. The effects of inspiratory airflow rates on deposition in the nasal turbinates were investigated using different airflow rates (0, 20, and 60 L/min) using methanol, water, and 60% glycerin formulations in a series of three different nasal spray devices [29]. It was concluded that there was no statistically significant change (*p* = 0.05, using one-way ANOVA), in turbinate deposition efficiency within each device/formulation when the airflow rate was increased from 0 to 60 L/min.

However, other studies incorporating adjunct airflow indicate that it could improve nasal drug transport by 2–3 cm towards the nasopharynx [30]. The complicated geometry of the nasal passages and the large intersubject variability could cause air turbulence that would ultimately affect deposition, and such a phenomenon has great importance in narrow nasal airways such as found in the pediatric population. The angle or orientation of spray delivery into the nasal cavity combined with higher airflow rates could also affect deposition patterns. At higher airflow rates, gravitational sedimentation is less significant, and particles become less inertial and more likely to be carried along with the airstream; some particles maintain their upward trajectory and most probably penetrate deeper towards the olfactory region when the inhalation airflow rate is high or they are ‘carried’ upwards.

**Figure 7:**
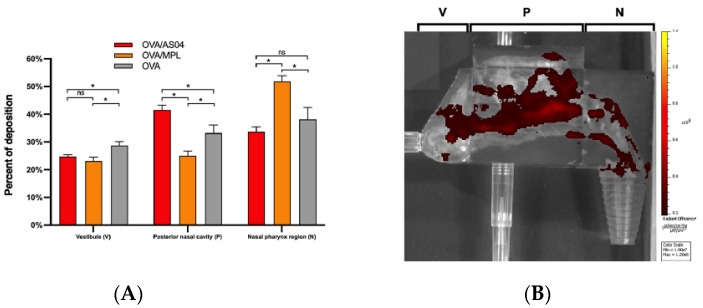
Intranasal deposition of three OVA antigen formulations in a nasal cast representative of a 7-year-old girl (**A**). The administration angle was 40° from the horizontal. Distribution (**B**) is shown in the vestibule (V), posterior region (P) and nasopharynx (N) from formulation OVA/AS04 [32] (ns: not significant, * *p* < 0.05).

Some studies have indicated that nasal deposition varies both within and between age groups [31]. With a growing interest in nasal vaccination, understanding deposition in children and pediatric patients is becoming more important. The deposition from three intranasal antigen formulations of ovalbumin (OVA) were compared using a nasal cast derived from a 7-year-old girl [32]. Deposition was quantified in three regions: the nasal vestibule, posterior nasal cavity and nasopharynx. The target for nasal vaccines is the nasal associated lymph tissue (NALT) located in the nasopharynx. As can be seen in Figure 7, the formulation containing the OVA antigen with the MPL (3-O-desacyl-4′-monophosphoryl lipid (A) adjuvant achieved a significant level of deposition in the region of interest when compared with the other formulations.

There has also been some interest in investigating in vitro the fraction of particles or droplets in nasal sprays <10 µm as these may pass through the nasal cavity and inadvertently be delivered to the lungs, which is not the intended target of nasal sprays. Several approaches have been used to measure the aerodynamic size of typical nasal sprays, including glass expansion chambers [33], and metal inlet ports with simplified geometry [34]. These approaches may give useful information about the fractions of the spray <10 µm but they do not come close to representing the nasal anatomy either in geometry or form as the glass expansion chambers are large circular 1L vessels, a volume much greater than typical human nasal volumes (mean: 26.4 cm^3^, range: 20.9–31.1 cm^3^ [19]). The metal nasal ports are angular tubes and do not closely represent the nasal cavity either. These kinds of in vitro approaches to investigating the percentage of fine fractions in nasal sprays are more suited to regulatory or quality control requirements.

**Figure 8:**
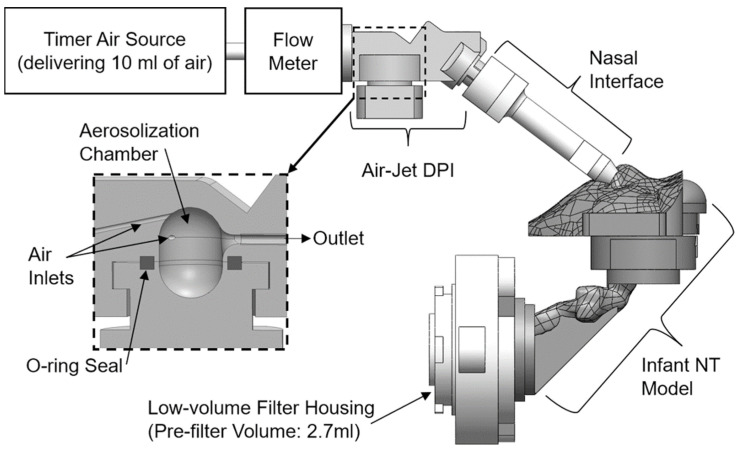
In vitro preterm infant nose throat (NT) model for evaluation of a novel in-line DPI. The inset shows an expanded view of the air-jet DPI, illustrating internal components, including the aerosolization chamber. NT, nose-throat [35].

Nasal casts may also be coupled with downstream measurements using, for example, a Next Generation Impactor to quantify the amount of drug that may deposit in the lung. Filters added after the nasopharynx region can also be utilized to estimate the inhaled dose. An example of such an experiment is shown in Figure 8 [35]. An infant nose throat model was developed to quantify nose to lung deposition of a trans-nasal in-line dry powder inhalation (DPI). Drug deposition in the nose-throat model and tracheal filter were quantified to compare different device designs. These studies illustrate the utility of in vitro models to evaluate device design.

As can been seen from the few examples above, nasal casts play a role in the development of intranasal drug products. Despite the obvious benefits, there are also drawbacks such an inability to quantify mucociliary clearance. Nasal casts are also not representative of entire populations. Nonetheless, many questions can be answered using these in vitro models.

## 5. Validating Nasal Casts: In Vitro vs. In Vivo

**Figure 9:**
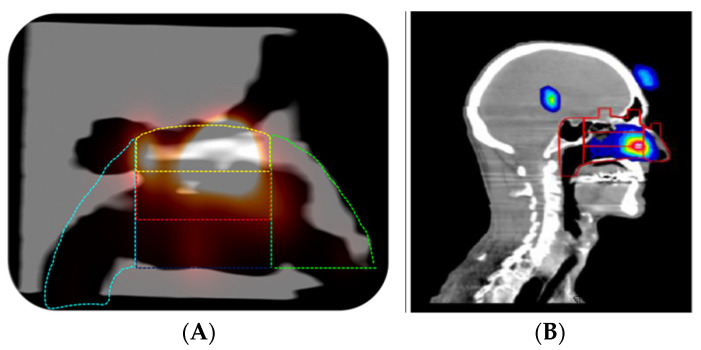
In vitro (**A**) vs. in vivo (**B**) deposition patterns as assessed by scintigraphy. The colour lines in the figure outline the regions of interest (ROI) in both the nasal cast (**A**) and human volunteers (**B**).

It is well documented that regional deposition of nasal sprays delivered to the nasal cavity is dependent on a large number of factors, including droplet size, velocity, spray orientation, spray cone angle, insertion depth, and nasal geometry [9], amongst others. Given that the anatomy can vary significantly, it is best practice to validate in vitro nasal cast models. This is often achieved by comparing in vitro deposition to in vivo deposition by gamma scintigraphy (Figure 9).

Various efforts have been made to build in vitro–in vivo correlations in the field of intranasal aerosols; they can be analyzed either in terms of the total amount of aerosol deposited in the nose or the regional deposition within the various sections of the nasal cavities. As there are no standard approaches for nasal casts, comparisons between studies can be difficult.

Only a few studies have investigated experimental set ups to formally test IVIVC implementing identical delivery and measurement approaches both in vitro and in vivo [36]. From such studies several key parameters have been identified which impact total nasal deposition efficiency: particle characteristics, ventilation and nasal anatomy.

Individual nasal anatomy has a strong influence on in vivo deposition and a study using a nasal spray on ten volunteers reported around 90% variability both in terms of deposition distribution between the upper/lower parts of the nasal cavities as well as the inner/outer nasal sections [37].

Studies investigating the effects of both nasal spray administration angle to the patient as well as anatomical variability have highlighted that nasal deposition targeting of the aerosol can be improved if patient specific approaches are used [25]. This involves developing individualized administration parameters for each patient and this may have particular value in emerging therapeutics when targeting specific regions (e.g., turbinates, olfactory region) and may prove critical with regard to efficacy of treatments indicated for life-threatening diseases as well as those with narrow therapeutic windows.

Employing realistic in vitro nasal replica models as described above, typically using computed tomography (CT) scans, often has the limitation that one can only study a given single subject or anatomy. An alternative approach is to develop an idealized nasal geometry that replicates average deposition for a population of subjects. This has been accomplished, and idealized nasal models are available in which CT scans for seven healthy individuals (five males, two females, with an average age of 60 years) were amalgamated using computational fluid dynamics (CFD). The idealized nasal model was segmented into the following regions: entrance, turbinates, olfactory and nasopharynx [25]. Good nasal deposition agreement was found when the idealized model was compared to 10 realistic replicas as well as to previously published regional deposition observed in vivo using gamma scintigraphy in five subjects, thus confirming this approach as a useful additional tool for nasal in vitro deposition testing.

Some researchers have proposed moving towards personalized medicine by printing nasal casts on-demand based on CT scans of patients [20]. 3D printing technology has evolved significantly on many levels including access, cost and rapidity and is now widely available in a variety of materials (transparent, biocompatible, and flexible). Therefore, one could envisage a scenario in which 3D scans from individual patients are used to manufacture personalized nasal cast models that are then used to optimize the drug delivery to these patients. This kind of approach could be beneficial where the therapies need to be accurately targeted in the nasal cavity in order to access the CNS, for example.

Additionally, nasal casts models can be combined with other techniques to collect complementary data. In this context, combining a nasal cast with a next generation impactor (NGI), for example, could give information about complete airway deposition for certain inhaled applications. Researchers have also combined in vitro RPMI2650 nasal cell culture models into a 3D printed model of the nose to test deposition and permeation of drugs intended for use in the nose [38]. Combining living nasal cell cultures with 3D printed casts in this way can give valuable information about several factors related to nasal drug delivery including drug deposition, permeation, dissolution and uptake. Future advances in this area will be central to advancing knowledge in nasal drug delivery.

## 6. Summary

In vitro nasal anatomical models have some known and recognized limitations including a lack of ability to represent aspects such as nasal mucosal surface variability and ciliary clearance (Table 1). Despite these shortcomings, nasal models have proved to be very useful tools and their validity has been verified by several techniques over the years such as acoustic rhinometry [19], gamma scintigraphy [23,38,39], CFD [22] and pharmacokinetics [20]. Based on these results together with the continuous improvement and evolution of nasal casts, in vitro studies involving nasal anatomical models will serve as useful tools for improving understanding in nasal drug delivery. They can be useful aids in various areas of research such as formulation development, nasal deposition of drugs, device development and optimization, opportunities for generic products and new avenues such as central nervous system (CNS) nasal drug delivery as well as other future innovations in this space.

In silico, CFD simulation or other promising developments may in future, with suitable validation, present additional approaches to current modelling, although the complexity and wide degree of variability in nasal anatomy will remain a challenge [26]. Currently, there seems to be no real in vitro substitute for in vivo assessment with regard to nasal deposition or performance, especially for regulatory requirements such as ‘bioequivalence’ for generics. This need is more pronounced for topical therapies where the nasal deposition patterns can strongly influence both safety and efficacy.

One disadvantage of using a nasal cast model to evaluate deposition is the challenge of standardizing or finding a ‘one size fits all’ model. Work needs to continue in order to build a range of models that are representative of different classes of patients, and perhaps one day to have an idealized model for each class.

Nevertheless, deposition studies using nasal casts appear to be a very helpful technique for comparing different nasal devices and various formulations. Moreover, they can provide key information around drug deposition in specific regions of the nasal cavity which may be key to the development of new nasal therapies. 

## Figures and Tables

**Table 1:** Pros and cons of nasal casts.

Pros	Cons
Can be validated against in vivo deposition	Does not reflect intersubject anatomical differences
Rapid screening method	Static with no mucociliary clearance
Facilitates device and formulation selection	No standardized model makes comparisons between laboratories difficult
Educational tool that illustrates deposition patterns	
